# An interpretative phenomenological analysis of lived experiences and psychological processes in internalized weight stigma

**DOI:** 10.1111/bjhp.12804

**Published:** 2025-05-14

**Authors:** Veronika Nagy, Lydia Poole, Esme Banting, Rose‐Marie Satherley

**Affiliations:** ^1^ School of Psychology, Department of Psychological Interventions, Faculty of Health and Medical Sciences University of Surrey Guildford UK; ^2^ Specialist Weight Management Service Ashford and St. Peter's Hospital NHS Foundation Trust Chertsey UK

**Keywords:** good health and wellbeing, obesity, qualitative, stigma

## Abstract

**Objective:**

Internalized weight stigma (IWS) refers to the internalization of societal weight‐based prejudices. While research on external weight stigma is well‐established, the psychological mechanisms underlying IWS remain underexplored. This study aims to provide a deeper understanding of IWS by examining the lived experiences of individuals with obesity and identifying key psychological processes contributing to IWS.

**Design:**

A qualitative design was employed, using both in‐depth interviews and photo‐elicitation to explore the lived experiences of nine participants.

**Methods:**

Participants were invited to take pictures of situations which made them feel stigmatized about their body size during a 2‐week‐long photography task. Subsequently, participants reflected on the implications of their photographs during a 60‐minute research interview. IPA was used to guide the analysis of the interview data.

**Results:**

Four key psychological processes contributing to IWS were identified: (1) Self‐application of negative stereotypes, where participants internalized societal stigma, sometimes resisting it but still experiencing self‐critical thoughts; (2) Imposition of an undesired identity, where societal labels restricted self‐expression, leading participants to adopt socially acceptable personas; (3) Heightened anxiety and social vigilance, where participants experienced anxiety, hyper‐awareness, and discomfort in public settings; and (4) Distress and coping, where emotional distress and coping strategies like social withdrawal appeared to reinforce IWS.

**Conclusions:**

These findings extended existing literature by providing a data‐driven conceptualization of IWS. The findings underscore the importance of developing psychological interventions that address both IWS and external societal weight stigma, focusing on strategies that challenge self‐critical narratives and promote more adaptive self‐concepts.

**Statement of Contribution**

**What is already known on this subject?**

- Internalized weight stigma (IWS) negatively impacts the mental health of individuals with obesity.
- Existing research primarily focuses on external weight stigma, overlooking internal psychological processes.
- Psychological mechanisms underlying IWS are underexplored.

**What does this study add?**

- Highlights the need for psychological interventions addressing both IWS and external societal weight stigma.
- Highlights the need for psychological interventions addressing both IWS and external societal weight stigma.

## BACKGROUND

Weight stigma, the societal stereotyping and devaluation of individuals in larger bodies, perpetuates harmful myths that weight is solely a matter of personal willpower (Crandall, 1994; Mata & Hertwig, 2018; Tomiyama, 2014). This stigma is prevalent in Western societies, where 40% of adults report that they have been treated unkindly due to their weight (Himmelstein et al., 2017; Puhl et al., 2021). Weight‐based stereotypes harm both the mental and physical health of those affected (Puhl & Suh, 2015; Tomiyama et al., 2018; Wu & Berry, 2018). Some develop internalized weight stigma (IWS), which involves devaluing oneself based on body weight and incorporating weight stigmatizing experiences into the self‐concept (Durso & Latner, 2008; Ratcliffe & Ellison, 2015). Research suggests that IWS is a stronger predictor of health outcomes than external experiences of weight stigma, serving as a potential mechanism through which weight stigma affects health (Latner et al., 2014; Pearl & Puhl, 2016).

While there is a positive association between weight stigma and IWS, not all individuals who experience stigma will internalize it (Pearl et al., 2018). Several factors that increase the likelihood of IWS have been identified; IWS is more likely in individuals who are White, low income, less educated, of higher body mass index (BMI), and interested in weight loss (Puhl et al., 2018). Furthermore, weight stigma experiences in familial or work settings may increase the likelihood of internalization (Pearl et al., 2018). Widely accepted models suggest that weight stigma is internalized via four sequential steps: awareness of stigma, agreement with these stigmatizing messages, self‐application of these stereotypes, and finally self‐devaluation (Corrigan et al., 2006; Pearl & Puhl, 2018). However, recent empirical evidence contradicts this model. For example, a recent mixed methods study suggested that some individuals could score highly on IWS measures without endorsing or applying weight‐based stereotypes to themselves (Pearl et al., 2023).

Recent reviews emphasize the need for qualitative research to explore the lived experiences of people in larger bodies who report experiencing weight stigma, focusing on the psychological processes they perceive as contributing to IWS (Romano et al., 2022). To address these gaps, the present qualitative study explored the psychological processes contributing to IWS in adults who self‐identify as overweight or obese and are engaged in weight management efforts. This person‐centred approach aims to advance conceptualizations of IWS by highlighting the nuanced, lived experiences of affected individuals.

## METHODS

The study design used photo‐elicitation (Harper, 2002) and semi‐structured interviews as the data collection methods, with interpretative phenomenological analysis (IPA) (Smith et al., 2021) to analyse IWS through participants' personal experiences. IPA is well‐suited for exploring emotionally significant topics, offering a framework to deeply explore personal meaning‐making processes. Complementing this, photo‐elicitation enabled participants to visually capture elements of their experiences. This approach encourages participants to reflect on their subjective meanings, leading to insights that may be less accessible through verbal questioning alone. This method empowers marginalized individuals by enabling them to identify the topics of significance, thereby reducing power imbalances in research (Catalani & Minkler, 2010; Puhl et al., 2008). While IPA principles were used to guide both data collection and analysis, photo‐elicitation was only utilized during the data collection phase to elicit rich accounts of participant experiences.

### Study context, eligibility and recruitment

This study was conducted in South East England. Participants were recruited, opportunistically, between January–July 2023 through social media sites (e.g., Facebook and Reddit), third sector organizations (e.g., Obesity UK and Diabetes UK), and a Specialist Weight Management Service in the National Health Service (NHS). Posters outlining the study's aims and procedures were distributed both physically and digitally following approval from gatekeepers. Participants were invited to express their interest in a study titled ‘*How Does Weight Stigma Impact Our Well‐being?*’ by providing their email address to the research team.

Eligible participants were adults aged 18 or older who self‐identified as overweight or obese, expressed an interest in weight management, and reported weight‐stigmatizing experiences. Additional requirements included access to a device capable of taking photographs and the ability to attend two online interviews. The study recruited a sample of nine participants, aligning with the methodological focus of IPA which favours in‐depth examination and case‐by‐case analysis (Smith et al., 2021). This sample size provided sufficient diversity to identify commonalities across participants' experiences while ensuring the idiographic depth essential to IPA's analytical approach.

### Data collection

Participants were invited to engage in a two‐phase procedure after receiving detailed written information about the study. All participants attended both phases on their own. The first phase involved a 30‐minute discussion conducted via Microsoft Teams with the researcher (VN). During this session, informed consent was obtained, rapport was established, and the photography task was explained. Participants were also informed that this research was being conducted as part of a doctoral qualification. Participants were instructed to spend the following 2 weeks taking photographs of objects, events, or situations that made them feel stigmatized due to their body size. To ensure privacy and safety, participants were advised to avoid capturing identifiable individuals or private locations (Hannes & Parylo, 2014). Logistical considerations, such as equipment requirements, time management, and secure submission of photographs, were also addressed. Following this initial discussion, participants independently completed the photography task over the next fortnight.

Two weeks later, participants took part in an audio‐recorded semi‐structured interview with the researcher (VN) via Microsoft Teams, lasting ~ 60‐minutes (see Supporting Information for interview guides). This interview, which was audio‐recorded, began with a discussion of their experience completing the photography task. Participants then shared selected photographs with the research team, which were displayed on screen to stimulate discussion. The photo‐elicitation process (Bartoli, 2020; Kirkham et al., 2015) included focused questions about specific images (e.g., ‘What do you see in this picture?’) that expanded into broader inquiries (e.g., ‘How does this shape who you are today?’). Following IPA's idiographic principles, the researcher acted as a ‘naïve listener’, allowing participants to lead the discussion and choose which photographs to emphasize (Smith et al., 2021). After the interview, all photographs were securely destroyed to uphold ethical standards.

### Analysis

Audio recordings of the interviews were transcribed verbatim by the lead researcher (VN). Analysis was carried out in Microsoft Word and then transferred to NVivo, using a systematic, iterative process that prioritized idiographic exploration before moving to more abstract themes. The analytical process began with one researcher (VN) reading each transcript multiple times to achieve immersion in the participant's account. Exploratory notes were made line‐by‐line, attending to the participants' explicit utterances, linguistic choices, and implicit meanings. To challenge preconceptions and encourage deeper engagement with the data, decontextualization techniques, such as reading the transcript backwards were employed until no further insights emerged.

Exploratory notes were then condensed into experiential statements summarizing participants' experiences in concise phrases. These statements were recorded in the margins alongside the corresponding data. All experiential statements for each participant were collated into a separate document and sorted into thematically similar clusters, reflecting both unique and shared elements of their lived experiences. The process then shifted to an across‐participant analysis, identifying convergence and divergence in themes while maintaining sensitivity to individual accounts. Salient themes were developed into group experiential themes, illustrated with verbatim quotes to preserve the richness of participants' voices. To uphold the trustworthiness and credibility of the analysis, discussions were held regularly within the research team throughout the interpretative process. These discussions allowed for diverse perspectives to be considered, enhancing the depth and rigour of the analysis, while ensuring the analysis remained grounded in the participants' accounts.

To uphold confidentiality, participants were assigned pseudonyms, and identifiable information was removed from transcripts. Photographs were excluded from the formal analysis to remain consistent with IPA's focus on participants' verbalized interpretations rather than visual data. However, it was felt that the use of photographs during interviews contributed to the richness of the verbal data that was obtained. While not systematically analysed, a recurring theme of tight spaces and objects (e.g., spiral staircases, park gates, small chairs), eateries (e.g., pubs, fast food restaurants), body parts (e.g., arms, stomachs), and social media images (e.g., lingerie models, screenshots of articles) was noticed. Participants generally found reviewing their photographs emotive, becoming tearful or mirthful upon their presentation. This heightened emotion may have aided state‐based recall, leading to specific and vivid descriptions of current and past experiences of stigma and internalized stigma. The photographs also helped to keep interviews focused, as discussions drifted toward generalities when photographs were not shown.

In keeping with the interpretative nature of IPA, a summary of findings was shared with participants to maintain transparency, though feedback on the findings was not sought. This was primarily because of IPA's hermeneutic position (Smith et al., 2021), which puts emphasis on the researchers' interpretation of participants' sense making within the analytical process.

Researcher reflexivity was integral to the analysis, supported by ongoing reflective discussions with the research team. This team comprised a trainee Clinical Psychologist (VN), two psychology academic researchers (LP, RS), and a Specialist Clinical Psychologist with expertise in NHS weight management services (EB). Reflexivity, a core element of IPA's double hermeneutic, ensured that the researcher critically engaged with their own assumptions and how these may influence the interpretative process.

### Ethical approval

The study received ethical approval from the South Central – Hampshire A Research Ethics Committee (Reference 22/SC/0375). This committee operates under the Health Research Authority in England.

## RESULTS

Nine participants completed the study, ranging in age from 25 to 65 years, with no dropouts. Of these, eight (88.9%) identified as female, and one as non‐binary. Most participants (88.9%) described their ethnicity as White British, with one participant identifying as Asian British. All participants self‐identified as obese, with body mass index (BMI) values in the corresponding category (Median = 44 kg/m^2^).

The analysis uncovered four key psychological processes contributing to IWS, encapsulated in four overarching themes (Table 1). These themes capture the nuanced ways participants made sense of their experiences, offering insight into the mechanisms underlying IWS. Subthemes are underlined throughout the text.

**Table 1:** Summary of themes and illustrative participant quotes.

Group experiential theme	Subtheme	Illustrative quotes
‘I'm fat and lazy’ – Self‐Application	Awareness of weight‐based judgement from others	*There was a lot of that still as a teenager, which I think is probably when things end up getting stuck more in your bright light (focus). If you hear it all the time, you know, ‘you've got thunder thighs, you got thighs like tree trunks’*, etcetera. *[Umm] It just sits with you, and it makes you not feel good about yourself*. **Jess** *After I ended that relationship than it, it became outright bullying. It it just it kind of became under the‐ I mean it's a slightly more targeted and piercing voice among the chorus of bullying… it got to me a lot, [Hmm] and I‐I think that the‐ the fact that it was not just coming from general classmates, it was also coming from my own partner*. **Harry**
	Applying others narratives to the self	*I mean, it's really easily done, isn't it, to believe other people's perception that, you know, you must go home and eat like six pizzas a night and etcetera, because of your size. And you know, ‘well your back's bad because you're fat’… And then when you look at people looking at fat people, you're thinking ‘Yeah, they're probably thinking something bad about that person’. Or ‘they're probably thinking something crap about me’. And I, you know, I get it, it's cause I'm fat. [Hmm] And then I'll probably order a doughnut with my coffee [chuckles]*. **Jess** *My grandson, the old one, he's like‐ he's poking me and he's going ‘You've got big muscles, haven't you?’. And I went ‘Oh they're not muscles’. [laugh] [laugh] Cuz they're not muscles. And he says, ‘Well, what is it?’ I goes ‘It's flub’. I goes ‘That's from eating too many sweets’ like trying to put him off*. *‘This is what happens if you eat too many sweets’*. **Rhea**
*‘You are just in this box’ –Ascribing to an Undesired Identity*	An imposed weight‐based identity that limited self‐expression	*But I mean, the whole big, beautiful woman thing, that's that that's a whole can of worms in its own right… Women, if you are a large woman, you are relegated to the big, beautiful woman category you you cannot appear in any other category [laugh] that you are, just know you are in this box…Doesn't matter what your other qualities are. You are just in this box… It's kind of othering… And you are reduced to a fetish*. **Harry** *But I think I do get that show on when I'm‐ I have to go out. [Pause] You know what I mean? But it's almost like, ‘well, if I'm bubbly and fat and jolly, then people, that's what people expect of me’. I've got to be jolly. I can't be. [Pause] [Hmm] In a mood or anything like that? Because big people aren't in a mood. They're fat and jolly. You know what I mean? So I've become that fat and jolly person so. [Pause] Yeah…But [my family] they almost know that the sort of like…the core‐ the first me, the me that I like, the me that‐, you know what I mean? I want to get back, to that kind of thing*. **Alice**
	Societal categories are ridged and impermeable	*It's really difficult because sometimes I think [Pause] there's almost a sense in my family that you live with what you've been given. So if you've been given a set of genes [Umm] that mean you're predisposed to put on weight then that's, you know, there's nothing you can do about that…*. *And I think there's this… kind of, ‘well, that's my genetic predisposition, therefore, you know that I can't fight against it’*. …*And in all honesty, I know it all, I know, I know what foods are better for me than other foods. I know what the right choices are. I I know what would make a difference. I think it's the motivation that's lacking, if I'm honest… So the fact that I could probably be a size 14 again and choose to wear whatever I wear, … but. it's like “oh, that will never happen to me because that I'm not a good enough person.”* **Simone** *So hopefully in the next 6–8 months I'm hoping to have weight loss surgery and [Pause] [Hmm] I suppose kick start back getting to the old me. [It will help me get] my confidence back again and a trying to recapture me from inside*. **Alice** *I'm hoping to you know, when I've lost a couple of more stone, be a bit more confident, like, you know, being on upright on it and stuff that I will get on that and I will join the human race. [Hmm] That's how I feel*. **Rhea**
‘*I worry constantly*’ – Anxiety Response	A vigilant awareness of weight stigma	*It doesn't feel natural. You are‐ I find myself constantly looking around thinking, you know‐ you know, ‘are they‐ are they staring?’ [laugh] And‐ ‘and if they're staring, why are they staring?’… That‐ that fear of judgement, of inviting mockery is‐ It in in a way it is disabling in its own right*. **Harry** *[At a party with a friend who wants to dance] And it was like “Come get up and dan‐* “*Sorry. “Get up‐ “Sorry. “Get up and dance!”. And I was like. “No, no, I'll just sit here and watch”…a lot of it is ‘I don't wanna be on the dance floor because if I'm on the dance floor, people are looking at me’ **Lisa*** *And it's SO stupid because I know that people aren't [looking and judging me]. They're not. [Hmm] But I think it's so ingrained into me that they look at me, they see a fat person….So like my rational‐ like my rational mind knows that that's probably not happening. But, where‐ it's kind of‐ it's been drilled into us that we're not good enough…And then again, that's‐ that whole‐ that paranoia kicks in*. **Ella**
	Self‐monitoring and self‐scrutiny in social situations	*[Visiting a historical manor] ‘Ohh stairs, wonderful, old stairs!’ And as soon as I saw them I would just see the immediate thought ‘Oh my God. Will those hold me? Will those hold me? Are they gonna break?’ Just the immediate irrational thought of these things are gonna give way under me*. **Harry** *That, you know, is a standard chair and you're kind of crammed into it, and you feel uncomfortable…You get really painful marks and things like that. But there's also that‐ there's definitely an emotional element of…You can't get comfortable. You feel like you're gonna need to move. That you're gonna look wrong in that chair as well… And I worry constantly*. **Jess**
‘*It's mentally exhausting’* – Distress and Coping	Profound emotional distress	*So feeling down, and I was diagnosed with severe depression about 3 years ago because obviously I felt‐ I honestly felt like ‘if I'd never woke from a coma, it would have done everybody a favour’, including myself, because I was trapped in this body that I hated*. **Alice** *A lot of the time I don't wanna be here. I don't feel I have a place in society. It's a huge issue for me. I'm scared for the future… Does that make sense? It's made me so scared and wary of people. Yeah. It's basically left me very socially isolated, to being almost close to hermit. Very much close to hermit*. **Sorcha**
	Social withdrawal	*So you know, there's that kind of‐ It's almost as if you project a stigma on to people and you reject. It's almost like you reject them first because like so that you, they can't reject you for being fat because you already rejected them*. **Simone** *I've sat at home and vegetated and felt very sorry for myself… Yes because in my own house, nobody can see me. …so I shut myself away and then I don't have to‐ Is it almost like a wall around myself that‐ it's a PHYSICAL wall as well as an emotional wall?… And I feel, I definitely do feel like I'm stuck in a shell…*. **Alice**

### Theme 1 – ‘I'm fat and lazy’ – Self‐application

This theme reflects how participants internalized societal stigma towards living with obesity, a psychological process known as self‐application (Corrigan et al., 2006). This process involved participants adopting societal attitudes about obesity as part of their self‐concept, often beginning in childhood when they became aware of weight‐based judgements from others. For many, these experiences marked the beginning of a self‐critical narrative shaped by the perceptions of others. Charlie reflected on how her sense of being ‘*different*’ first emerged in adolescence, when comments about her size during puberty began to shape her self‐concept. She noted, ‘*basically everything has been other people's perceptions on myself*’, indicating that external judgements, rather than her own self‐image, were the driving force behind her IWS. This illustrates how external societal messages shaped her psychological experience:

When I was a child, I liked my body. I never really thought about it… It was mostly more in puberty, and it was more to do with other people's comments where I was like ‘Ohh. Maybe my body is different’ …So yeah, basically everything has‐ has been other people's perceptions on myself. And then that's kind of related to then my‐ my own relationship with my weight. Charlie

Similarly, Harry described a particularly painful moment when a childhood boyfriend made a remark about their weight. This comment stood out as a ‘*more targeted and piercing voice among the chorus of bullying*’, with the metaphor of ‘*piercing*’ suggesting a deeper emotional wound than the more generalized societal judgements. For Harry, the remark from someone in an intimate relationship seemed to carry greater emotional weight, illustrating how close, trusted relationships can make the internalization of weight stigma more profound. Jess also highlighted how weight‐shaming comments from her mother, originating in childhood, transformed into a self‐critical voice in adulthood. Jess described how these critical messages became ingrained in her internal dialogue, continuously influencing her behaviour and thoughts.

It was a big thing for my mum, about not getting fat… you know, ‘you don't want to be fat’… ‘you're getting a bit chubby’… now there's almost that voice in the back of my head, kind of constantly telling me ‘you need to eat healthy, you need to eat healthy.’ And it's almost like well, you know, she's still telling me whether I like it or not. Jess

Childhood did not feature prominently in every participant's account. Rhea and Alice emphasized how their awareness of weight‐based judgements emerged in adulthood, following life‐changing disabilities which impacted their body size.

During the interviews, participants were applying the judgemental narratives of others to the self. For instance, Rhea attributed her weight to ‘*eating too many sweets*’, echoing a message she had been told in childhood. Alice repeatedly used the words ‘*fat*’ and ‘*lazy*’ to describe herself, reflecting a deeply ingrained and rigid self‐criticism. Jess explicitly acknowledged her sensitivity to societal perceptions, stating that it was ‘*easily done*’ to assume others thought she must ‘*go home and eat like six pizzas a night*.’ She further expressed, ‘*if [others were] thinking something crap about me, [she would] get it—it's ‘cos I'm fat*’, capturing how external judgements diminished her self‐esteem and reinforced feelings of unworthiness. On the other hand, Sorcha resisted applying judgemental narratives to herself, firmly locating the problem in society. She attributed her ability to do this to education and engagement with social justice initiatives.

I'd get taken in for coffee or a tea and sat down, ‘You've gained a lot of weight recently Sorcha, have you thought about eating more salad or this or that?’ And you're just sitting there going ‘I need to get out of this room’ … people don't understand that weight gain has so many aspects … the lack of education is the problem. Sorcha

### Theme 2 – ‘You are just in this box’ –ascribing to an undesired identity

This theme describes how participants experienced being confined to a set of undesirable, externally imposed identities due to their weight. Participants spoke about an imposed weight‐based identity that limited self‐expression and defined how they were perceived in social interactions. This ascribed identity was described as restrictive, with many participants feeling trapped within labels that did not reflect their true selves. The language used to describe this experience was often reductive and dehumanizing, as illustrated by Harry:

The whole big, beautiful woman thing, that's that that's a whole can of worms in its own right… if you are a large woman, you are relegated to the big, beautiful woman category you you cannot appear in any other category [laugh] that you are, just know you are in this box…Doesn't matter what your other qualities are. You are just in this box… It's kind of othering… And you are reduced to a fetish. Harry

For Harry, being categorized as a ‘*big, beautiful woman*’ felt limiting, reducing them to a single aspect of their identity and leaving little room for other qualities. This sense of confinement was echoed by Ella who expressed that being one's ‘*own person is not desirable*’, indicating that her authentic identity was often inaccessible under the weight‐based label imposed by society. Many participants struggled to envision what their authentic self would look like outside the constraints of this imposed identity. Alice, for instance, referred to her authentic self in the abstract, possibly due to a lack of space or opportunity to explore identities beyond her socially ascribed one. Alice described the pressure to perform a ‘*bubbly and fat and jolly*’ persona that others expected from her, while distancing herself from her ‘*core—the first me, the me that I like*.’ The act of masking or suppressing her true self to conform to external expectations appeared to reinforce feelings of disconnection and unworthiness.

On the other hand, Simone described taking on the identity of a ‘feminist’ in her 30s, which helped her to reject the idea of *‘femininity*’, and the societal value placed on body image. Unlike other participants Simone seems to have found this identity empowering in her youth, as it allowed her to direct her difficult feelings around her body image outwards rather than inwards. Notably, Simone came to see the feminist identity of her 30s as confining in hindsight, sharing how it precluded her from doing some of the things that she loved.

With me it [body image]‐ it's very intrinsically linked to my‐ my idea of what‐ what a feminist is…I think part of me decided that it was not being not feminist to wear makeup, whereas of course now I love wearing lip lipstick in particular…I think that kind of like, you know, making yourself look feminine was I really rejected that whole idea. [Mm‐hmm] [Umm] Because I thought it was serving men rather than serving my own interest…Whereas now I kind of don't worry about what the world thinks, [Mm‐hmm] I mean, quite so much. Simone

The notion that societal categories are rigid and impermeable emerged strongly across participants' accounts. For some, weight loss efforts became an attempt to escape these identities. Lisa's reflected on a period of weight loss, recalling how this had allowed her to *‘recapture me from inside’* enabling her to access a version of herself not tied to her body size. She described a moment of liberation during her school prom, where she was recognized as *‘the blonde*’, an identity that felt disconnected from her weight. Rhea also described weight loss as a way of ‘*join*[ing] *the human race*’, implying that losing weight would help her gain a sense of belonging and confidence:

I'm hoping to you know, when I've lost a couple of more stone, be a bit more confident, like, you know, being on upright on it and stuff that I will get on that and I will join the human race. [Hmm] That's how I feel. Rhea

However, for most participants, attempts at transformation were brief, often followed by the realization that their bodies—and the identities tied to them—could not be permanently changed. Alice, for example, described her resignation upon regaining weight, saying she was ‘*destined to be fat*.’ Simone echoed this sentiment, feeling as though she couldn't ‘*fight against*’ her body size. These reflections reveal a cyclical nature of self‐ascribed stigma, where the inability to permanently alter their bodies reinforced feelings of being trapped within an undesired identity.

### Theme 3 – ‘*I worry constantly*’ – Anxiety response

This theme explores the heightened anxiety participants experienced as they navigated social environments. They exhibited a vigilant awareness of weight stigma, anticipating judgement or negative evaluation based on their size. Many participants felt scrutinized in public spaces, which contributed to a sense of vulnerability. Harry described this constant state of alertness, noting they were ‘*constantly looking around*’, while Ella articulated the sense that ‘*everybody's looking at me. Everybody's judging me*.’ These reflections capture the attunement to others' gaze, which was often interpreted negatively, even in the absence of direct evidence of judgement. For Rhea, the expectation of judgement was so ingrained that she felt as though society could only see her as a ‘*big person struggling*.’ She expressed a belief that others were not capable of seeing her in a positive light:

And that's all they see is a big person struggling…. I don't think they'll look at you in a positive light. Nobody said ‘Oh, that's a lovely fat person there’. You know, ‘That's a beautiful person’, you know. They'll look at your shape rather than your personality… So they're looking down on you. Rhea

Rhea's account emphasizes the self‐perception of being othered due to her size and reinforces the emotional weight of societal stigma. The automatic, and visceral reaction to weight‐based judgement was further illustrated by Sorcha, who described her heightened vigilance as a ‘*horrible instinctive gut reaction*.’ This constant awareness of stigma became so pervasive that even neutral or positive interactions were scrutinized, often through a lens of suspicion. Sorcha reflected on how she would doubt the sincerity of compliments, interpreting them with caution:

I'm on the hunt for ‘What's the agenda? What's behind it? What do they want? What's coming next?’ … I have that horrible instinctive gut reaction now … If my husband pays me a compliment, and he's really kind about it, he might say like ‘That colour suits you’ or something. Immediately, I'm like well, ‘Don't say that. Don't talk to me. Can't cope with that. Don't want compliments that don't fit. Sorcha

Some participants, like Alice, were able to distinguish between what they knew to be true and what they felt internally. Alice reflected on the tension between her ‘*level head’*, which did not believe others were staring, and the ‘*messed‐up brain*’ that made her feel intensely observed. This tension between perception and reality added complexity to participants' experiences of anxiety, illustrating the internal conflict between their cognitive awareness and emotional reactions.

In my level head, I don't believe they're staring at me, but in my messed‐up brain, I feel it intensely. Alice

This disconnect between what participants consciously knew to be true and the emotional response they had suggests the deeply internalized nature of weight stigma, which could not be easily dismissed by rational thought.

An equally significant psychological process was self‐monitoring and self‐scrutiny in social situations. Participants reported feeling intensely self‐conscious about their bodies, often scrutinizing themselves in public and internalizing societal perceptions of their appearance. Rhea, for instance, recounted her experience at her daughter's wedding, where instead of enjoying the event, she focused on how her body felt: ‘*bloated, fat, swollen*.’ This shift from experiencing the moment to bodily self‐monitoring reinforced her sense of discomfort and inadequacy. Jess similarly described how her body felt in a camping chair, likening herself to a ‘marshmallow’ with ‘*bumps coming out on the sides*.’ This vivid imagery reflected how participants externalized their self‐view, as if perceiving their bodies through the critical gaze of others, rather than their own perspective:

It's like a marshmallow squeezed into a seat. And all the bumps were kind of coming out on the sides. And it just makes you feel like people are gonna judge you for how you look when you're, like, sat out in in the park on a chair like that. Jess

For Jess, the externalized perception of her body intensified her feelings of discomfort and led her to scan her surroundings for judgement, highlighting how internal self‐scrutiny and external vigilance reinforced one another. This dynamic of self‐monitoring was also noted by Alice, who observed that her feelings of inadequacy could cause her to ‘*possibly portray that onto other people*’, speaking to the interplay between internal evaluations of the self and perceived external judgements. This demonstrates how they could mutually reinforce and perpetuate the experience of IWS.

### Theme 4 – ‘*It's mentally exhausting’* – Distress and coping

Participants described profound emotional distress associated with living with obesity, including feelings of sadness, hopelessness, and depression. For Alice, distress was deeply rooted in her altered self‐identity following weight gain caused by a physical disability. She described feeling *‘trapped in this body that I hated*’, expressing a profound sense of disconnection from her changed physical form, which she now viewed as foreign and restrictive. For some participants, this emotional pain escalated to suicidal ideation, as Sorcha revealed when she shared, ‘*a lot of the time I don't wanna be here. I don't feel I have a place in society*,’ reflecting a sense of alienation and hopelessness.

Participants consistently identified habitual self‐monitoring and vigilance as key contributors to emotional exhaustion and typically used social withdrawal to cope. Sorcha spoke of avoiding others when ‘*feeling down*’ and Lisa recounted retreating to her bed, feeling unable to face others. For Alice, this emotional distress created a sense of immobilization, as she described feeling trapped behind both a physical and emotional wall, preventing her from pursuing desired changes:

In my own house, nobody can see me. …so I shut myself away and then I don't have to‐ Is it almost like a wall around myself that‐ it's a PHYSICAL wall as well as an emotional wall. Alice

This withdrawal provided a form of protection from external judgement but also reinforced feelings of isolation and sadness, maintaining participants' distress. By isolating themselves, they limited opportunities for positive interactions that could challenge their IWS and reshape their perceptions of how others viewed them. Simone highlighted this cyclical pattern of avoidance when she shared how, in her younger years, she would pre‐emptively reject others due to fears of being rejected herself. Later, with increased confidence, Simone engaged more openly with others and discovered that her earlier fears of rejection were ‘*almost imagined*’, suggesting that greater social engagement could transform negative assumptions:

I would reject them first. But later, when I became more confident, I found that those fears were almost imagined. **Simone**

Other participants, like Harry, described strategies to mitigate their distress in social settings. Harry explained that they would carefully layer clothing to hide their body, avoid eating in public to prevent judgements of being ‘*greedy*’, and limit social interactions to close, trusted friends. These coping strategies provided temporary relief but also reinforced their IWS, restricting their social participation and reinforcing feelings of self‐consciousness. Harry described the societal pressure to always present a perfect image:

I'm always dressed nicely…I can't go out feeling like I look unkempt… the fact is, if you're a bigger person, you get judged. And you are judged more harshly. And you know if if you go out wearing something that's not immaculate as a big person, you invite all other kinds of criticism about you having let yourself go or your slovenly, this, that, the other, and it stops just being a comment on your weight and it almost becomes a moral judgement. Harry

Harry's reflection demonstrates how societal judgements extend beyond the physical and become moralized, contributing to exhaustion and reinforcing participants' struggles with IWS.

## DISCUSSION

Despite the growing recognition of IWS and its harmful effects on health and well‐being, much of the existing literature has predominantly focused on the external dimensions of weight stigma, such as societal discrimination and public shaming. By contrast, this study prioritizes the lived experiences of participants, offering valuable insights into the internal processes individuals undergo facing IWS. This phenomenological approach allows for a deeper understanding of how individuals navigate weight stigma from their own perspectives, revealing nuanced psychological mechanisms such as the internalization of stigmatizing narratives, the imposition of an undesired identity, the emergence of anxiety responses, and the management of distress through social withdrawal.

Central to understanding IWS is the process of self‐application of negative stereotypes. While Corrigan et al.'s (2006) Stage Model of Self‐Stigma suggests a linear process involving awareness, agreement, and self‐application of stigma, the experiences of participants in this study indicate a more nuanced process. Participants demonstrated a clear awareness of societal judgements about body size, recognizing stereotypes directed at themselves and others. However, they did not uniformly agree with these stereotypes, suggesting that internalization of stigma is not always a straightforward or passive process. This divergence from Corrigan's model points to a more intricate interplay of cognitive processes. Participants seemed to both acknowledge and resist societal judgements, often recognizing the stigmatization aimed at others but still internalizing the negativity directed at themselves. This finding resonates with the work of Pearl et al. (2023), which suggests that individuals may resist external stereotypes and yet still experience the internalization of stigma. Such complexity underscores the need to consider the individual's cognitive appraisal and personal history in understanding how weight stigma becomes internalized, further challenging linear models of self‐stigma.

One of the most striking findings of this study is how participants were forced into a rigid weight‐based identity, which often felt alienating and restrictive. This may align s with the concept of masking, as described by Hull et al. (2017). In their work, masking refers to a process where neurodivergent individuals enact socially acceptable behaviours to avoid stigma. Similarly, participants in this study spoke of adopting a ‘*bubbly and jolly*’ persona or engaging in roles dictated by societal expectations of overweight individuals. This act of social camouflage, where participants perform an identity that is not truly their own, may contribute to the perpetuation of IWS by reinforcing feelings of unworthiness and internalizing the belief that they must conform to external expectations to be accepted. Interestingly, these findings may suggest that the internal struggle of holding an undesired identity—even when such an identity might offer social acceptance—can still contribute to IWS. This contrasts with existing literature, which typically emphasizes the external societal threats posed by weight stigma (Major & O'Brien, 2005). Instead, this study highlights that the internalization of a weight‐based identity could be a source of psychological distress, operating independently of external judgements. This may underscore a more complex and multifaceted relationship between IWS and self‐concept, where internal struggles complicate how individuals perceive and navigate their identities, beyond simply reacting to societal judgement.

Participants also described heightened anxiety and vigilance related to social scrutiny, indicating that anxiety is a common and recurring experience in IWS. This aligns with previous research suggesting that the awareness of weight stigma keeps it at the forefront of individuals' minds, thereby facilitating its incorporation into the self‐concept (Hunger et al., 2020). The present findings may also resonate with the Social Anxiety Model (Clark & Wells, 1995), wherein individuals perceive themselves as social objects (e.g., Rhea referring to herself as an *‘alien’*), and rely on internal cues to gauge external judgements. Thus, the participants' internal focus may exacerbate anxiety and anticipated judgement regarding their body size, suggesting a bi‐directional relationship between IWS and socially focused anxiety.

**FIGURE 1:**
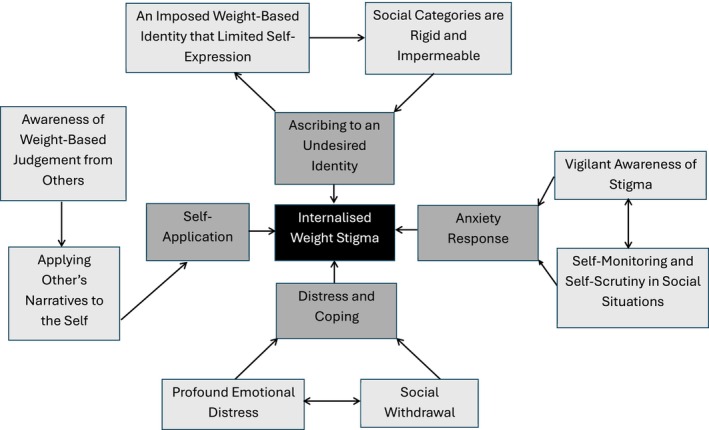
A proposed model of the psychological process involved in the formation of internalized weight stigma.

The findings in Theme 4 (Distress and Coping) underscore the emotional toll of IWS. The apparent cumulative effect of constant scrutiny and negative self‐perception may contribute to an ongoing cycle of distress and social withdrawal. Participants' accounts of withdrawal were consistent with depression, where social withdrawal is thought to interact with other symptoms such as diminished activity and rumination to maintain an individual's difficulties (Papworth & Marrinan, 2018). For instance, some participants described how their heightened vigilance resulted in avoidance behaviours that prevented them from engaging in social situations, reinforcing their feelings of isolation and inadequacy. Indeed, social withdrawal may maintain distress by preventing individuals from disconfirming their negative cognitive biases (Papworth & Marrinan, 2018). Safety behaviours (such as social withdrawal) in the context of living with obesity may be unique, as certain fears around being stigmatized may be grounded in the reality of the societal context (Puhl & Heuer, 2009). However, in such instances, safety behaviours may still maintain distress and unhelpful beliefs around an individual's ability to cope. In turn, this may contribute to IWS, as an individual may start to devalue themselves due to their perception that they are a weak person for not coping with ordinary situations (Ratcliffe & Ellison, 2015). Combined, our findings have led us to develop a schematic figure of the psychological processes underlying the development of IWS (see Figure 1).

Current interventions aimed at IWS primarily draw from cognitive behavioural therapy and acceptance and commitment therapy, focusing on increasing psychological flexibility and adherence to health‐promoting behaviours (Levin et al., 2018; Palmeira et al., 2017; Pearl et al., 2020). While these approaches offer support for socially focused anxiety and challenges related to undesired identities—through skills like valued action, defusion, distress tolerance, and self‐acceptance—they often do not explicitly address these concerns. Given the study's tentative identification of social anxiety and the ascription to an undesired identity as significant contributors to IWS, future interventions may benefit from directly targeting these processes to enhance their effectiveness.

### Strengths and limitations

A key strength of this study was its phenomenological and idiographic approach, which enabled the identification of novel contributors to IWS directly from participants' lived experiences. However, the study's cross‐sectional qualitative design limited the ability to confirm the strength and directionality of relationships among identified factors. Further work is therefore needed to test our proposed model using alternative methods. In addition, the current study did not explore the psychological processes that facilitate resistance to IWS, an important area for future research that could inform strength‐based interventions to prevent/minimize IWS. While all participants in this study exhibited IWS, previous research suggests that belonging to a stigmatized group does not necessarily result in self‐devaluation (Crocker & Major, 1989). The paradox model of self‐stigma indicates that individuals who view stigma against their group as illegitimate can employ various cognitive, emotional, and behavioural strategies to resist it, ultimately leading to higher global self‐esteem (Corrigan & Watson, 2002).

The current study had a small and relatively homogeneous sample, largely consisting of White British women of working age, which means the intersection of different identities and IWS could not be considered. Previous research has shown that men and women have different responses to weight stigma: while women may develop a negative perception of their body size, prompting attempts to lose weight through restrained eating (Wellman et al., 2018), men may view experiences of weight stigma as emasculating and respond by asserting their masculinity in other ways such as growing a beard (Lozano‐Sufrategui et al., 2016). These gendered responses point to the potential influence of demographic factors on the experience and expression of IWS. As such, our findings may not be generalizable to more diverse populations.

### Conclusion

The current study extended existing literature by providing a broader conceptualization of IWS comprised of four related psychological processes: self‐application, anxiety, distress, and the ascription to an undesired ‘fat identity’. These findings could be used to scaffold the design of more refined assessment and treatment tools and to adapt existing psychological interventions to more effectively reduce IWS in those living with obesity.

## AUTHOR CONTRIBUTIONS

**Veronika Nagy:** Conceptualization; investigation; writing – original draft; methodology; writing – review and editing; formal analysis; data curation. **Lydia Poole:** Formal analysis; writing – review and editing; project administration; supervision; software. **Esme Banting:** Conceptualization; writing – review and editing; formal analysis; supervision. **Rose‐Marie Satherley:** Conceptualization; investigation; methodology; writing – review and editing; software; project administration; formal analysis; supervision.

## FUNDING INFORMATION

The authors declare that no funding was received for this research.

## CONFLICT OF INTEREST STATEMENT

There are no conflicts of interest to declare.

## Supporting information


Data S1:

## Data Availability

Anonymized data supporting the findings of this study are available upon reasonable request from the corresponding author. Due to the sensitive nature of the study, participant’ consent was obtained under the understanding that data would remain anonymous. Therefore, raw data will not be made publicly available to ensure participant anonymity and privacy.
