# Supplementary material for: An interpretative phenomenological analysis of lived experiences and psychological processes in internalized weight stigma
Source: Br J Health Psychol. 2025 May 14;30(2):e12804. doi: 10.1111/bjhp.12804 (PMC12078877; doi:10.1111/bjhp.12804)
Supplement: Supplementary file 1 — Data S1: [file BJHP-30-0-s001.docx]

**Supporting Information. Preparatory Interview Guide**

Introduction:

- Introduce yourself as the researcher and explain your role in the study.
- Provide an overview of the study, referencing the information sheet and consent form. Emphasise the participant’s right to withdraw at any time without consequence.
- Briefly describe the aims of this interview: to set up the photography task.
- Obtain verbal consent from the participant to proceed.
- Invite any questions before proceeding.

*Note: This interview will not be recorded.*

Topic #1: Assessing Participant’s Expectations for the Project

Aim:

- To assess if the participant’s expectations align with the study’s focus and to help them reflect on their engagement with the project.

Key Questions:

1. What about the recruitment material for this project interested you?
2. Why do you want to be involved in this project?
3. How do you understand weight stigma, and how do you think it might show up in daily life?
4. Have you done any photography before, or is this something you’d enjoy doing?

Topic #2: The Photography Task

Aim:

- To ensure the participant understands the exercise and feels comfortable with it.

Information Sharing:

- In the next two weeks, you will be asked to take photos of anything that makes you feel negatively about your body weight.

Key Questions:

1. Can you think of something from the past week that you could have taken a picture of that made you feel bad about your body weight?
2. Why would you have chosen this specific object or situation to photograph?

Topic #3: Taking Pictures Safely and Respectfully

Aim:

- To ensure the participant considers privacy and safety when taking pictures.

Information Sharing:

- Respect the privacy of others while taking photos, especially in public spaces.
- Do not photograph identifiable individuals or their private spaces (e.g., homes, workplaces).
- It's acceptable for people to appear in pictures, provided they are not the sole focus, are in large groups, and their faces are not visible.
- Take care of your own safety when taking photos (e.g., avoid standing in dangerous places, such as in the road).

Key Question:

- How could you capture your experiences without including identifiable people or specific private spaces? (Allow participant to brainstorm alternative ideas).

Topic #4: Logistics

Aim:

- To help the participant plan how and when to take their photos.

Information Sharing:

- Taking part in this project can be time-consuming, so it’s helpful to plan ahead. Consider these points for the next two weeks:

Questions:

1. Do you have access to a camera or smartphone to take the photos?
2. Do you feel you have enough time and mental space in the next two weeks to dedicate to this project?
3. When might be a good time for you to take your photos?
4. Is there anything else we should consider in planning for the next two weeks?

Topic #5: Sending Pictures to the Researcher and Interview Scheduling

Aim:

- To clarify what happens after the participant has taken their photos.

Information Sharing:

- After you’ve taken the photos, send them to me using the Surrey University Secure File Transfer Link. We will review them in our next interview.
- Your photos will be stored confidentially..
- Reiterate the consent process and participant’s right to withdraw at any time.

Questions:

1. When would you like to schedule our next meeting to discuss your photos?

Ending:

Summary Question:

- How prepared do you feel to complete the photovoice project?

Final Question:

- Is there anything we should have discussed that we didn’t?

**Semi-structured interview guide**

Introduction Recap:

- Reintroduce yourself and your role.
- Recap the study purpose and the right to withdraw.
- State the aim of the interview: to explore how the participant makes sense of their photos and the impact on their experience of weight stigma.
- Reconfirm verbal consent.
- Ask if there are any questions before beginning.

Photo Discussion:

S: What do you see in this picture?

- Encourage the participant to describe the image and its initial thoughts.

H: What’s happening in the picture?

- Ask the participant to explain the situation, context, or story behind the photo.

O: How does this relate to your personal experience?

- Explore how this photo connects to their life, feelings, or perceptions of weight stigma.

Present Reflection:

Impact on Self/Behaviour:

- Did this photo affect how you think about yourself or your behaviours?
- How has it influenced your views on your body or wellbeing?

Impact on Others/Society:

- How does this image relate to your perceptions of how others or society view weight and body size?

Past Reflection:

Previous Experiences:

- Have you had similar experiences in the past that this photo brings up for you?
- How did these experiences shape your beliefs or self-identity?

Why:

Origins of Stigma:

- Why do you think weight stigma exists?
- Where do you think these ideas about body size and weight come from?
- How do societal, familial, or healthcare reactions contribute to these beliefs?

Uncaptured Experiences:

- Are there other experiences related to weight stigma that you haven’t been able to capture in your photos? How have those affected you?

Education:

Lessons for Others:

- How can this image help others understand weight stigma and its impact?
- What do you want people to take away from your photos?

Action:

What Can Be Done:

- What should society or healthcare systems do more or less of to address weight stigma?
- Have you experienced weight stigma in healthcare settings? How did that affect your care or health outcomes?

Consent:

- Confirm consent for using photos in the final write-up. Allow opt-out.

Ending:

Summary:

- How well do you feel this discussion captures the impact of weight stigma on long-term well-being?

Final Question:

- Is there anything else you would like to add or discuss?
